# Entomological surveillance and spatiotemporal risk assessment of sand fly-borne diseases in Cyprus

**DOI:** 10.1016/j.crpvbd.2023.100152

**Published:** 2023-11-06

**Authors:** Maria Christou, Behich Koyutourk, Kardelen Yetismis, Angeliki F. Martinou, Vasiliki Christodoulou, Maria Koliou, Maria Antoniou, Christoforos Pavlou, Yusuf Ozbel, Ozge Erisoz Kasap, Bulent Alten, Pantelis Georgiades, George K. Georgiou, Theodoros Christoudias, Yiannis Proestos, Jos Lelieveld, Kamil Erguler

**Affiliations:** a The Cyprus Institute, Nicosia, Cyprus; b Joint Services Health Unit, British Forces Cyprus, RAF Akrotiri, Akrotiri, BFPO 57, Cyprus; c Department of Parasitology, Ege University, Institute of Health Science, Izmir, Turkey; d Veterinary Services, Ministry of Agriculture, Nicosia, Cyprus; e Medical School, University of Cyprus, Nicosia, Cyprus; f University of Crete, Crete, Greece; g Department of Parasitology, Ege University, Izmir, Turkey; h Max Planck Institute for Chemistry, Mainz, Germany; i Biology Department, VERG Laboratories, Hacettepe University, Beytepe-Ankara, Turkey

**Keywords:** Leishmaniasis, *Phlebotomus papatasi*, Habitat suitability, Population dynamics, Climate-sensitive, Vector-borne diseases

## Abstract

Visceral and cutaneous leishmaniases are important public health concerns in Cyprus. Although the diseases, historically prevalent on the island, were nearly eradicated by 1996, an increase in frequency and geographical spread has recently been recorded. Upward trends in leishmaniasis prevalence have largely been attributed to environmental changes that amplify the abundance and activity of its vector, the phlebotomine sand flies. Here, we performed an extensive field study across the island to map the sand fly fauna and compared the presence and distribution of the species found with historical records. We mapped the habitat preferences of *Phlebotomus papatasi* and *P. tobbi*, two medically important species, and predicted the seasonal abundance of *P. papatasi* at unprecedented spatiotemporal resolution using a climate-sensitive population dynamics model driven by high-resolution meteorological forecasting. Our compendium holds a record of 18 species and the locations of a subset, including those of potential public and veterinary health concern. We confirmed that *P. papatasi* is widespread, especially in densely urbanized areas, and predicted that its abundance uniformly peaks across the island at the end of summer. We identified potential hotspots of *P. papatasi* activity even after this peak. Our results form a foundation to inform public health planning and contribute to the development of effective, efficient, and environmentally sensitive strategies to control sand fly populations and prevent sand fly-borne diseases.

## Highlights

• The first georeferenced database of sand fly species in Cyprus.
• The first model-based island-wide seasonal profiling of *Phlebotomus papatasi.*
• Links meteorological covariates and land type with sand fly physiology.
• Sand fly activity occurs in multiple peaks but almost uniformly across the island.
• Highlights hotspots and times for planning sand fly control.

## Graphical abstract

**Figure:**
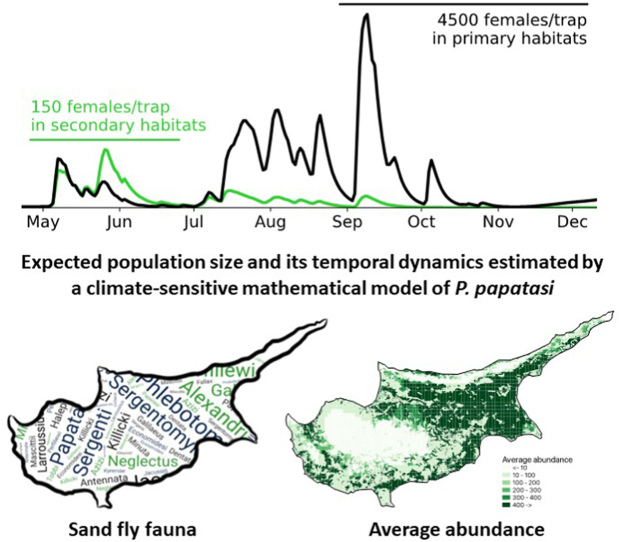

## 1 Introduction

Neglected tropical diseases (NTD) are a diverse group of communicable diseases that occur in tropical and subtropical regions and affect more than one billion people globally (CDC, 2021). Leishmaniasis is classified as an NTD, caused by parasites of the genus *Leishmania* (pathogens), which are transmitted to humans and animals (hosts) through infected female phlebotomine sand flies (vectors). The Eastern Mediterranean Region accounts for 80% of the cutaneous leishmaniasis cases worldwide (WHO, 2021). The most serious form and often fatal, also called kala-azar, is attributed to vector species of the genus of *Phlebotomus* in the Old World, and of *Lutzomyia* in the New World. The main foci of Old World leishmaniasis are located in China, India, Central Asia, East Africa, the Mediterranean basin, and Brazil (Dedet, 2010).

Environmental changes such as deforestation, building of dams, irrigation schemes, and urbanization have been directly linked to leishmaniasis infections (WHO, 2021). Global warming is among the most important drivers of the potential expansion of leishmaniases, while tourism and trade could facilitate the transportation of its vectors and pathogens all over the globe (Dujardin et al., 2008). Although *Phlebotomus* sand flies are unable to actively disperse over distant areas (Léger et al., 2000b), environmental changes may provide suitable conditions for their survival and reproduction and promote their geographical expansion (Medlock et al., 2014).

Cyprus, an island between Southeast Europe, North Africa, and West Asia, is prone to the transmission of leishmaniasis due to a list of factors, including urbanization, extensive agriculture, changing environmental conditions, and population movement from countries where the disease is endemic (Antoniou et al., 2008). *Phlebotomus* sand flies are widespread in Cyprus, and there is evidence of pathogenicity among Cypriot patients (Alwassouf et al., 2016; Billioud et al., 2019). Although the visceral and cutaneous forms of leishmaniasis were nearly eradicated by 1996, recent evidence suggests an increase in frequency among the population due to the active circulation of the parasites transmitted by autochthonous sand fly species and a set of favourable conditions potentially leading to their geographical spread (Mazeris et al., 2010). Cyprus is the only area in Europe where cases of anthroponotic visceral leishmaniasis have been reported and attributed to *Leishmania donovani*, which is related to *Leishmania infantum*, while an *L. infantum/L. donovani* hybrid has been reported in *Phlebotomus tobbi* (Antoniou et al., 2008; Seblova et al., 2015).

Field inventories and geodatabases of vector species are essential tools for public health planning. A comprehensive account of vector fauna, however, is often a challenge as individual studies can each cover small parts of a region. Here, we sampled and compiled the data about sand fly fauna across Cyprus and investigated the potential distributions of a selected subset - selected for species importance in disease transmission. We employed remote sensing datasets and weather-driven population dynamics modelling to bridge the gaps and derive high-resolution geospatial and seasonal activity. Applications as such demonstrate the use of predictive mathematical modelling in studying leishmaniasis epidemiology and hold the promise of identifying long-term changes in geographical patterns due to climate change.

## 2 Materials and methods

### 2.1 Geographical distribution of sand fly species in Cyprus

Sand fly sampling was performed intermittently between 2013 and 2020 through the collaboration of three institutions: The University of Crete, the Joint Services Health Unitʼs Vector Ecology and Applied Entomology Laboratory, and Ege University. Adult specimens were collected from 8 villages, located in the southwest and central areas of the island, with CDC miniature light traps equipped with a fine net cage, and morphologically identified using published keys (Lewis, 1987).

The list of sand fly species was complemented with peer-reviewed literature on sand fly ecology in Cyprus. The terms “sand flies”, “phlebotomine”, “leishmaniasis”, and “Cyprus” were screened in English, Greek, Turkish, and French using Google Scholar, the University of Illinois Library, and Scopus. References mentioned within the relevant publications were also examined; however, studies reporting pathogens or leishmaniasis infections were excluded.

Geographical coordinates, where available, or the city/village of the sampling locations were mapped using the publicly available QGIS 3.14 software.

### 2.2 Land cover preferences of the species of medical importance

Land cover preference was assessed for two sand fly species, *Phlebotomus papatasi* and *P. tobbi*, based on expert opinion retrieved from ECDC (2019). The preference scale is based on CORINE Land Cover (CLC) classification and employs 3 levels: (i) primary land type (land classes providing the most suitable habitat for a species and providing the likelihood of the greatest vector numbers); (ii) secondary land type (land classes where a species may still be found but less likely and in much lower numbers than above); and (iii) unsuitable land type (land classes where a species is unlikely to be found except in exceptional circumstances). The CLC classification for 2018 was retrieved from the Copernicus Land Monitoring Service and used for preference mapping (Kosztra et al., 2019).

### 2.3 Generation of high-resolution meteorological covariates

Meteorological data were generated over Cyprus for the year 2015 using the open-source, community-based, state-of-the-art Weather Research and Forecasting (WRF-ARW) Model (Skamarock et al., 2008). The model was configured according to the operational numerical weather forecasts of the Cyprus Department of Meteorology. The model set-up has been extensively validated and proven highly accurate for the Eastern Mediterranean and Cyprus (Georgiou et al., 2018). The meteorological fields, generated with a nested configuration setup, were at an ultra-fine spatiotemporal resolution (i.e. 2 km horizontal grid spacing and 1-h temporal frequency).

### 2.4 Spatiotemporal modelling of sand fly abundance

The expected population size of *P. papatasi* in Cyprus in 2015 was simulated using the stochastic climate-driven population dynamics model of the species presented in Erguler et al. (2019). The model was simulated with air temperature and relative humidity obtained from the meteorological model. Two sets of parameters, labelled Combined A for Steni and Combined A for Geri - each with 1000 alternative configurations - were used to simulate the average number of adult females per day per trap (a proxy to expected population size). Model output implies that the system is observed similarly - the sampling design is identical - throughout the island and is the same as in the original publication. The first three months of 2015 were discarded as the transient phase of the simulations.

The two parameter sets, inferred for the Steni and Geri villages, are identical in physiological traits and environmental dependencies except for initial conditions and the fraction Ψ_B_. The fraction represents the impact of breeding site conditions on fecundity, gonotrophic cycle, and larva and pupa development. As evident from the land type classification and the original publication, observations were made mainly on the primary land type in Steni and on the secondary land type in Geri. Thus, population size was simulated over the island by switching between the two sets for each land type.

We present in Supplementary Text S1 a Python code to perform the spatiotemporal simulations described.

## 3 Results and discussion

### 3.1 Exploring the boundaries of the rich sand fly fauna in Cyprus

**Fig. 1:**
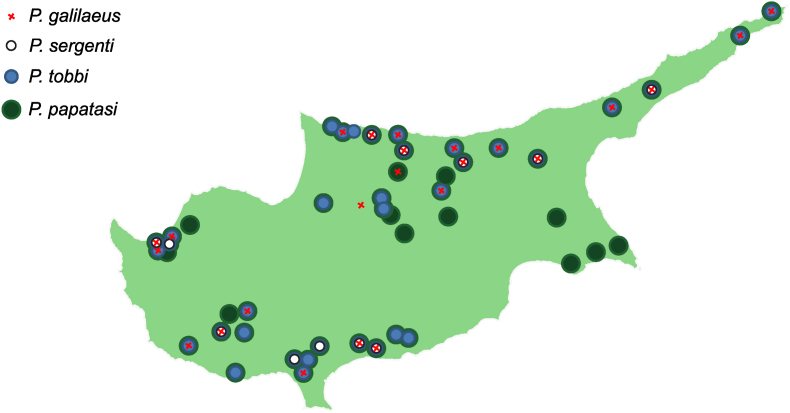
The distribution of the four common sand fly species in Cyprus. The marks represent the locations of the centroids of the administrative regions where the species were detected.

We compiled a dataset of sand fly presence in Cyprus with 18 species of phlebotomine sand flies from 2 genera and 6 subgenera. A list of all species is given in Table 1 and maps for species with known geolocations are provided in Supplementary Figs. S1–S3. We found that *P. papatasi*, *P. tobbi*, *P. sergenti*, and *P. galilaeus* are among the most frequently reported and widely distributed species; their locations are mapped in Fig. 1. While *P. papatasi* was found in almost all sampling locations, *P. tobbi* exhibited a slightly narrower range (not recorded in the south-eastern peninsula). On the contrary, *P. mascittii* (3 locations), *P. kyreniae* (5 locations), and *P. killicki* (1 location) have been seldom encountered. We note that, due to the morphological similarity within the subgenus *Transphlebotomus*, the presence of *P. mascittii* on the island has been found suspicious and in need of further molecular confirmation (Kasap et al., 2015).

**Table 1:** The compendium of the sand fly species of Cyprus, 1946–2023.

Subgenus	Species	References															
		1	2	3	4	5	6	7	8	9	10	11	12	13	14	15	16
*Phlebotomus*	*Phlebotomus papatasi*	✓	✓	✓		✓	✓	✓	✓	✓	✓	✓	✓		✓	✓	✓
*Artemievus*	*Phlebotomus alexandri*	✓		✓		✓		✓	✓	✓	✓						✓
*Paraphlebotomus*	*Phlebotomus sergenti*	✓	✓	✓		✓	✓	✓	✓	✓	✓		✓				✓
	*Phlebotomus jacusieli*					✓		✓	✓			✓					
*Larroussius*	*Phlebotomus perfiliewi*	✓		✓								✓		✓			✓
	*Phlebotomus tobbi*	✓	✓	✓		✓	✓	✓	✓	✓	✓	✓	✓	✓		✓	✓
	*Phlebotomus galilaeus*	✓	✓			✓	✓	✓	✓	✓	✓		✓				✓
	*Phlebotomus neglectus*							✓	✓				✓				✓
	*Larroussius* sp.											✓					
*Adlerius*	*Phlebotomus halepensis*							✓									
	*Phlebotomus kyreniae*	✓						✓	✓		✓						
*Transphlebotomus*	*Phlebotomus killicki*														✓		
	*Phlebotomus economidesi*				✓		✓	✓	✓	✓							
	*Phlebotomus mascittii*	✓				✓				✓							
*Sergentomyia*	*Sergentomyia minuta*	✓	✓	✓		✓	✓	✓	✓		✓	✓			✓		✓
	*Sergentomyia azizi*	✓	✓			✓	✓	✓	✓		✓	✓					
	*Sergentomyia fallax*	✓	✓	✓		✓	✓	✓	✓		✓						✓
	*Sergentomyia dentata*			✓											✓		✓
	*Sergentomyia antennata*	✓	✓														
	*Sergentomyia* sp.											✓	✓				✓
	Unidentified species			✓													✓

*References*: 1, Adler (1946); 2, Minter and Eitrem (1989); 3, Field et al. (1999); 4, Léger et al. (2000a); 5, Léger et al. (2000b); 6, Depaquit et al. (2001); 7, Rastgeldi et al. (2005); 8, Demir et al. (2010); 9, Mazeris et al. (2010); 10, Töz et al. (2013); 11, Ergunay et al. (2014); 12, Alten et al. (2016); 13, Dokianakis et al. (2016); 14, Dokianakis et al. (2018); 15, Erguler et al. (2019); 16, Present study.

Through our survey, we confirmed the presence of 7 species of *Phlebotomus* and 3 species of *Sergentomya* (Supplementary Table S1 and Supplementary Figs. S1–S3). As expected, *P. papatasi* was the dominant species, in all of the locations, followed by *P. tobbi* and *P. galilaeus*, albeit fewer in numbers. In addition, we detected *S. minuta* in large numbers in Aigoi Trimithias, which suggests that the village is a novel hotspot for this species. We did not detect certain species, such as *P. kyreniae* and *P. economidesi*, which exhibited restricted geographical ranges. Our dataset suggests that the number of species identified largely depends on the geographical extent of the study design. For instance, the surveys reported by Demir et al. (2010), Léger et al. (2000b), and Töz et al. (2013), differ largely from those by Dokianakis et al. (2016, 2018) and Erguler et al. (2019), with respect to the area covered and the number of species reported.

We note that while the earlier reports, including the comprehensive assessments of Adler (1946) and Minter and Eitrem (1989), employed morphological identification methods; contemporary reports employed genetic and serology techniques more often as a result of the recent developments in biochemical and molecular analysis (Field et al., 1999; Ergunay et al., 2014; Dokianakis et al., 2016, 2018). In addition to the improved accuracy in identification, molecular methods enable establishing phylogenetic relationships between populations at different locations.

*Phlebotomus papatasi* and *P. perfiliewi* are common vectors of sand fly fever viruses and likely causes of phlebovirus circulation in the Cypriot population (Alwassouf et al., 2016; Billioud et al., 2019). *Phlebotomus tobbi* has been related to infections with cutaneous and visceral leishmaniasis in the Middle East and the Eastern Mediterranean basin (Seblova et al., 2015). Likewise, *P. papatasi* and *P. sergenti* have been identified as vectors of *Leishmania major* and *L. tropica*, respectively (Volf and Myskova, 2007). Due to suitable rodent reservoirs for the parasite, *L. major* has long been acknowledged as endemic in the Jordan Valley even though it is currently missing from Europe (Özbilgin et al., 2019). In the last few decades, however, cases caused by *L. major* were reported outside its endemic range, such as in the southern region of Israel, i.e. Negev highlands, the western Negev, the Arava (Özbilgin et al., 2019), and south-eastern Turkey, i.e. Adana Province (Saroufim et al., 2014).

### 3.2 Risk assessment: Mapping sand fly abundance in space and time

**Fig. 2:**
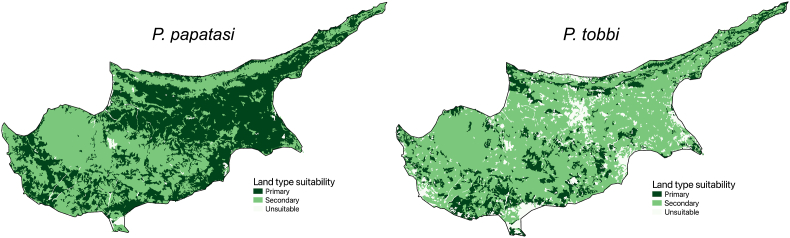
Land type preference for *P. papatasi* and *P. tobbi*.

To bridge the gap between observations, we used expert assessment on habitat preferences applied on satellite-derived high-resolution land cover data (Kosztra et al., 2019). The key for suitable habitat types, i.e. primary, secondary, and unsuitable habitat types, is published in the recent technical report of ECDC for a range of vector species (ECDC, 2019). We used this key to map the habitat preferences of *P. papatasi* and *P. tobbi*, two of the most abundant and medically important sand fly species, in Cyprus (Fig. 2).

We found that the primary habitats of *P. papatasi* are urban and suburban areas while *P. tobbi* is well-adjusted to Mediterranean sclerophyllous vegetation - a secondary habitat for *P. papatasi*. Rural locations of Cyprus typically include low scrublands/phrygana and maqui vegetation, which can affect sand fly diversity and thus infection risk. In previous studies, the broad-leaved forest was emphasized as a suitable habitat for sand flies (Martinez et al., 2007); however, this kind of habitat only makes up a small portion of the island.

The widespread distribution of *P. papatasi*, predicted by its habitat preference, is highly consistent with the observations and is a result of its strong ecological adaptability. This species has been observed in high densities in damaged ecosystems and has been collected from a variety of biotopes (Wasserberg et al., 2003; Guernaoui and Boumezzough, 2009). It also adapts well to artificial environments (Kamhawi et al., 1991).

**Fig. 3:**
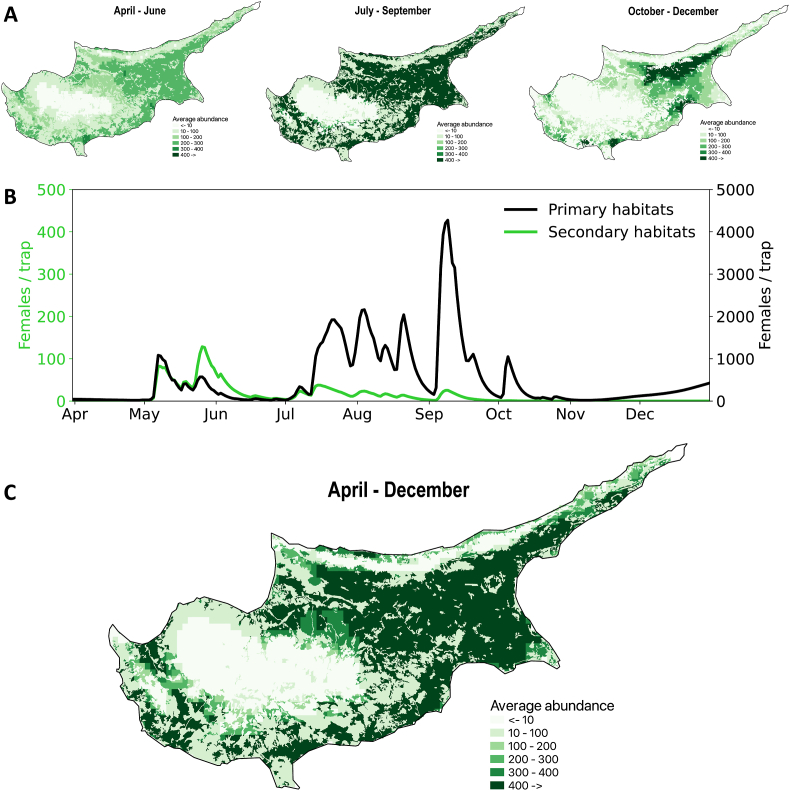
The expected population size of *P. papatasi* estimated by climate-sensitive mathematical modelling. Geospatial distribution of abundance is shown in panel **A** for three time periods (April-June, July–September, and October-December), and matches the temporal dynamics (**B**). The temporal dynamics of average number of females is given for both primary (*black line*) and secondary (*green line*) habitats. The average geospatial distribution of population size is given in panel **C**, where abundance corresponds to the average number of females per day per trap (see Section 2.4).

In addition to the availability of appropriate breeding grounds, we employed expected population size, estimated by climate-sensitive mathematical modelling, as a proxy to disease risk due to *P. papatasi*. We simulated the average number of adult females (see Section 2.4) from April to December (Fig. 3C), and found that it matches the observed distribution of the species on the island (Fig. 1). In particular, *P. papatasi* is absent from areas of high altitude and maintains high numbers in densely populated urban and suburban areas with high levels of recorded temperature and relative humidity. The highest abundance was predicted along the southern coastline and the Mesaoria Plain, including the capital of the island, Nicosia.

We identified multiple peaks of activity throughout the year starting in May and ending in September, when the population gradually declines (Fig. 3A). Two distinct periods of activity emerged and corresponded to two main generations of sand flies. The first generation appears in May and slowly disappears in June, from when the second, more sustained generation follows. We note that the second generation appears not in isolation but is a combination of two or three overlapping generations maintained by the suitability of ambient temperature and near-surface relative humidity.

We found that *P. papatasi* population size increases approximately uniformly across the island, except around the Troodos Mountain, until it peaks in July-September (Fig. 3A). The peak season is displaced with relatively lower and more clustered abundance in October-December (Fig. 3A), where high population size is maintained in certain areas, “hotspots”, such as Avdimou, Limassol, Larnaca, Nicosia, and the northern section of the Mesaoria Plain.

The spatiotemporal dynamics agrees well with the surveillance reports from counties with similar climates, such as Greece (Tsirigotakis et al., 2018) and Israel (Müller et al., 2011). In Israel, many sand fly species concentrate in humid areas during dry summers and reach their peak numbers at the end of the summer period, similar to the July-September period identified in Cyprus. In April and May, when vegetation is thick and relative humidity is high, many species tend to distribute evenly throughout their habitats (Müller et al., 2011), similar to the April-June period identified in Cyprus.

Here, we included a range of biotic, e.g. population structure and climate-sensitive physiology, and abiotic factors, e.g. temperature, relative humidity, and land cover, to predict the dynamics of *P. papatasi*. In addition, we note that socioeconomic factors, the population of stray dogs, certain types of land cover (e.g. dump sites, quarries, green urban areas, and vineyards), and altitude are also important factors for sand fly populations and leishmaniasis spread (Artun and Kavur, 2017; Iliopoulou et al., 2018).

Although the population of stray dogs is directly linked with canine leishmaniasis infections, the possibility of human infections typically increases with the number of infected dogs in an area (Mazeris et al., 2010). We plan to incorporate these additional factors, as well as the dynamics of pathogen reservoirs and disease transmission, in future studies for a more in-depth assessment of risk.

We established a direct link between a meteorological model and a population dynamics model, predicting the dynamics of *P. papatasi* across the island on a daily basis. Although we concluded our analysis in a calendar year, our setup enables executing the WRF model in forecasting mode and extending predictions into the future for short-term operational risk assessment.

## 4 Conclusions

Leishmaniasis is a climate-sensitive disease; temperature, land cover, and relative humidity have profound impacts on the ecology of its vector, phlebotomine sand flies, and thus the intensity of the vector-host-parasite interactions. Cyprus hosts a rich sand fly fauna and an active circulation of *Leishmania* parasites, which pose both veterinary and public health concerns. The island-wide distribution patterns, composed for several species, naturally exhibit gaps and observational biases. Here, we showed that climate-sensitive mathematical modelling, assimilated with satellite imagery and meteorological models, augments observations to improve our understanding of the spatiotemporal dynamics of selected species. Model-based risk assessment can indicate potential breeding habitats and times of peak activity, informing public health policies for developing optimum intervention strategies. The risk of sand fly-borne disease outbreaks can thus be reduced by employing site-specific measures rather than using area-wide applications of pesticides, therefore, minimising the environmental impact of vector control.

## Funding

This research did not receive any specific grant from funding agencies in the public, commercial, or not-for-profit sectors. This research was performed within the framework of the EMME-CARE project, which received funding from the European Unionʼs 10.13039/100010661Horizon 2020 Research and Innovation Programme under grant agreement No. 856612 and the Cyprus Government. The funders had no role in the design of the study; in the collection, analyses, or interpretation of data; in the writing of the manuscript, or in the decision to publish the results.

## Ethical approval

Not applicable.

## CRediT authorship contribution statement

**Maria Christou:** Conceptualization, Formal analysis, Investigation, Data curation, Visualization, Writing – original draft, Writing – review & editing. **Behich Koyutourk:** Investigation, Data curation, Visualization, Writing – review & editing. **Kardelen Yetismis:** Investigation, Writing – review & editing. **Angeliki F. Martinou:** Investigation, Resources, Writing – review & editing. **Vasiliki Christodoulou:** Investigation, Writing – review & editing. **Maria Koliou:** Supervision, Writing – review & editing. **Maria Antoniou:** Resources, Supervision, Writing – review & editing. **Christoforos Pavlou:** Investigation, Writing – review & editing. **Yusuf Ozbel:** Resources, Supervision, Writing – review & editing. **Ozge Erisoz Kasap:** Data curation, Writing – review & editing. **Bulent Alten:** Resources, Supervision, Writing – review & editing. **Pantelis Georgiades:** Investigation, Writing – review & editing. **George K. Georgiou:** Investigation, Writing – review & editing. **Theodoros Christoudias:** Investigation, Supervision, Writing – review & editing. **Yiannis Proestos:** Investigation, Writing – review & editing. **Jos Lelieveld:** Resources, Supervision, Writing – review & editing. **Kamil Erguler:** Conceptualization, Methodology, Software, Formal analysis, Visualization, Project administration, Supervision, Writing – review & editing, All authors have read and approved the final version of the manuscript.

## Declaration of competing interests

The authors declare that they have no known competing financial interests or personal relationships that could have appeared to influence the work reported in this paper.

## Data Availability

The data supporting the conclusions of this article are included within the article and its supplementary files. The entomological surveillance data are available in Table 1 and Supplementary Table S1. Python code to perform the spatiotemporal simulations described is provided in Supplementary Text S1. The meteorological covariates are available at https://doi.org/10.5281/zenodo.8413232, and the spatiotemporal simulation outputs are available at https://doi.org/10.5281/zenodo.8413593.
