# Supplementary material for: Entomological surveillance and spatiotemporal risk assessment of sand fly-borne diseases in Cyprus
Source: Curr Res Parasitol Vector Borne Dis. 2023 Nov 6;4:100152. doi: 10.1016/j.crpvbd.2023.100152 (PMC10787173; doi:10.1016/j.crpvbd.2023.100152)
Supplement: Multimedia component 1 [file mmc1.pdf]

Supplementary Figures S1-S3

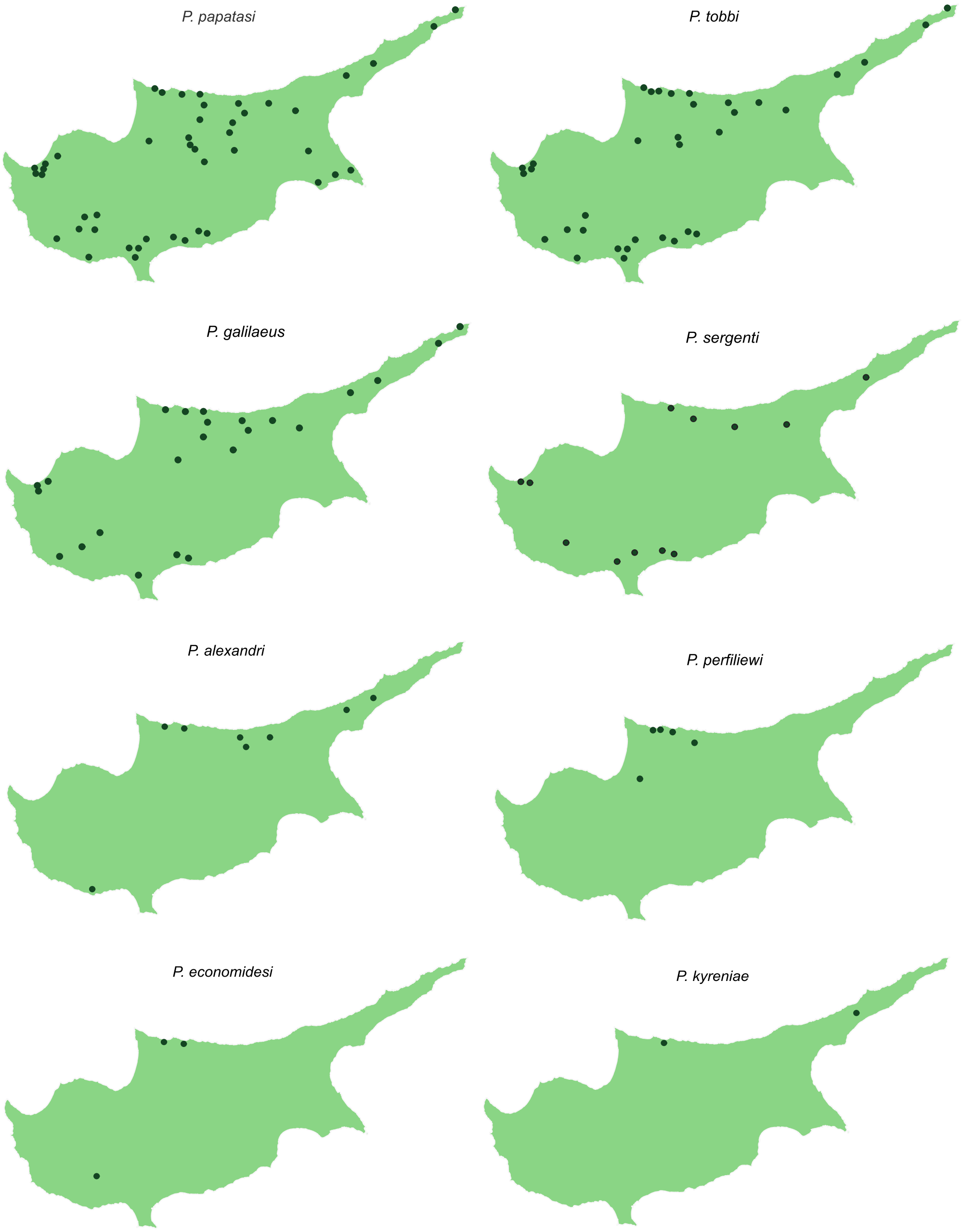

Supplementary Figure S1. Records of phlebotomine subspecies in Cyprus between 1946 and 2023. The marks represent the locations of the centroids of the administrative regions where the species are detected.

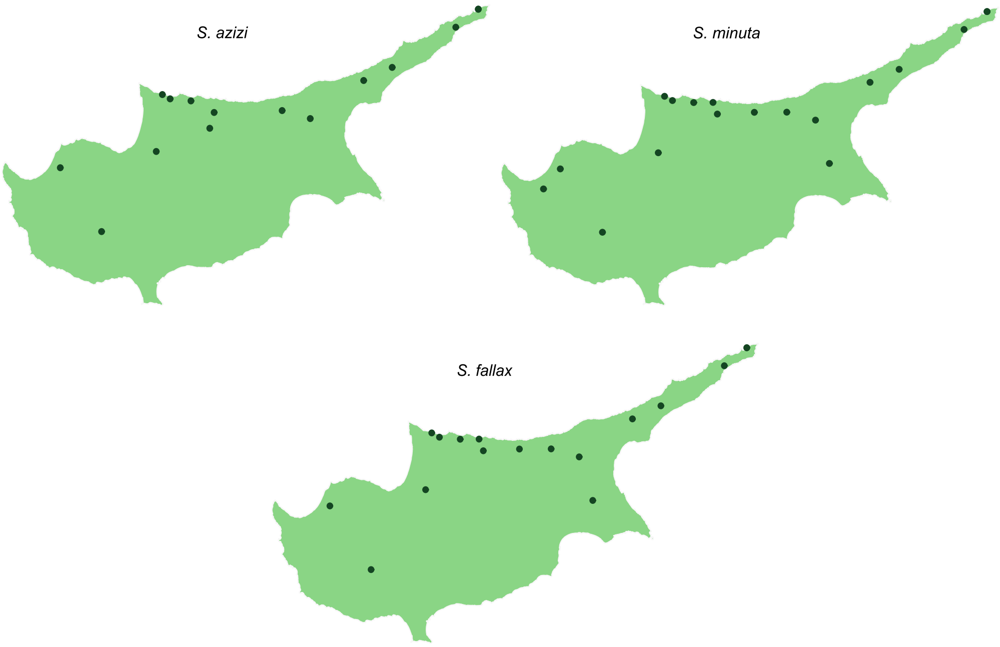

Supplementary Figure S2. Records of *Sergentomyia* subspecies in Cyprus between 1946 and 2023. The marks represent the locations of the centroids of the administrative regions where the species are detected.

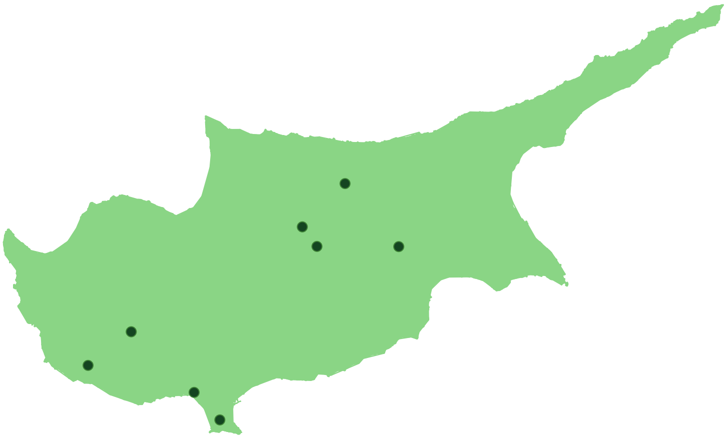

Supplementary Figure S3. Sand fly sampling locations.
