# Supplementary material for: Entomological surveillance and spatiotemporal risk assessment of sand fly-borne diseases in Cyprus
Source: Curr Res Parasitol Vector Borne Dis. 2023 Nov 6;4:100152. doi: 10.1016/j.crpvbd.2023.100152 (PMC10787173; doi:10.1016/j.crpvbd.2023.100152)
Supplement: Multimedia component 2 [file mmc2.pdf]

Supplementary Text S1

The Python code to perform the simulations described in Methods (2.3).

```
### Python Code File for CRPVBD Manuscript
### Entomological surveillance and spatiotemporal risk assessment
### of sand fly-borne diseases in Cyprus
###
### Copyright (C) 2023  Kamil Erguler
###
###     This program is free software: you can redistribute it
###     and/or modify it under the terms of the GNU General
###     Public License as published by the Free Software Foundation,
###     version 3 of the License.
###
###     This program is distributed in the hope that it will be useful,
###     but WITHOUT ANY WARRANTY; without even the implied warranty of
###     MERCHANTABILITY or FITNESS FOR A PARTICULAR PURPOSE.  See the
###     GNU General Public License for more details
###     (<https://www.gnu.org/licenses/>).

import numpy
import xarray
import albopictus as aa

# Uncomment the following to perform simulations
# over primary habitats
location = 'STENI'
# Uncomment the following to perform simulations
# over secondary habitats
# location = 'GERI'

# Adult female vectors should be extracted from model output
key = 'colnvAf'
```

```
# The model and parameter configurations are available in
# the albopictus package
model = aa.sand
parmat = numpy.array(aa.param['sand'][location]['papatasi']['combinedA']).copy()

# The meteorological datasets are available at
# Zenodo (10.5281/zenodo.8413232)
ds_T2 =
xarray.open_dataset("wrf_met_daily_avg_2015_curvfix_rlonlat_remapycon_NCL_T2_corine-
grid_rbil_r0.0215_2.nc")
ds_RH =
xarray.open_dataset("wrf_met_daily_avg_2015_curvfix_rlonlat_remapycon_NCL_RH2_corine-
grid_rbil_r0.0215_2.nc")
lons = ds_T2.lon.values[:]
lats = ds_T2.lat.values[:]
latiloni = numpy.array([[lat,lon] for lat in range(len(lats)) for lon in range(len(lons))])
mat_T2 = ds_T2.T2.values[:,:,:]
mat_RH = ds_RH.RH2.values[:,:,:]

# Prepare the matrix to store the model output
sims = numpy.ndarray((365,lats.shape[0],lons.shape[0]),dtype=numpy.float64)
sims[:,:,:] = numpy.nan

# The following routine prepares the environmental data
# for a given grid cell
def prepare(lati,loni):
    return {
        'dates': numpy.arange(mat_T2.shape[0]),
        'temp': mat_T2[:,lati,loni],
        'RH': mat_RH[:,lati,loni] * 0.01,
        'envar':
            numpy.hstack([
                mat_T2[:,lati,loni],
                mat_RH[:,lati,loni] * 0.01,
                numpy.repeat(0, mat_T2.shape[0])
```

```
        ])
    }

# The following routine performs a simulation for a given
# meteorological dataset (temperature and relative humidity)
def simClim(clim,pr,key='colegg'):
    rmean = []
    for clm in clim:
        if pr.ndim==1:
            r = model.simPar(clm,pr)
        else:
            r = model.simParMat(clm,pr,boil={key:[]})
        if (r.__class__.__name__!='dict' or not r['success']):
            return []
        if len(rmean)==0:
            rmean = numpy.array(r[key],ndmin=2)
        else:
            rmean = numpy.vstack([rmean,r[key]])
    return numpy.mean(rmean,axis=0)

# The following loop initiates the simulation for each grid cell
# available in the meteorological datasets
for lati,loni in latiloni:
    clim = prepare(lati,loni)
    if not numpy.all(numpy.isnan(clim['temp'])):
        print("Processing (%g,%g)" %(lons[loni],lats[lati]))
        #
        sim = simClim([clim],parmat,key=key)
        sims[:,lati,loni] = sim

# The output is available at Zenodo (10.5281/zenodo.8413593)
filename = "mech_model_STENI_papatasi_combinedA_%s_posterior_mean_%s.npy"
%(key,location)
numpy.save(filename, sims)
```
