# Supplementary material for: Entomological surveillance and spatiotemporal risk assessment of sand fly-borne diseases in Cyprus
Source: Curr Res Parasitol Vector Borne Dis. 2023 Nov 6;4:100152. doi: 10.1016/j.crpvbd.2023.100152 (PMC10787173; doi:10.1016/j.crpvbd.2023.100152)
Supplement: Multimedia component 3 [file mmc3.pdf]

Supplementary Table S1. Results of the sand fly sampling performed between 2013 and 2020 through an inter-institutional collaboration.

						Phlebotomus	*Artemievus*	*Paraphlebotomus*		*Larroussiuscc*					*Adlerius*		*Transphlebotomus*			*Sergentomyia*					
						Phlebotomus papatasi	*Phlebotomus alexandri*	*Phlebotomus sergenti*	*Phlebotomus jacusieli*	*Phlebotomus perfiliewi*	*Phlebotomus tobbi*	*Phlebotomus gallilaeus*	*Phlebotomus neglectus*	*Larroussius sp.*	*Phlebotomus halepensis*	*Phlebotomus kyreniae*	*Phlebotomus killicki*	*Phlebotomus economidesi*	*Phlebotomus mascittii*	*Sergentomyia minuta*	*Sergentomyia azizi*	*Sergentomyia fallax*	*Sergentomyia dentata*	*Sergentomyia antennata*	*Sergentomyia sp.*
**Group**	**Location**	**Longitude**	**Latitude**	**Year**	**Total**	**456**	**2**	**11**		**3**	**50**	**64**	**1**							**291**		**35**	**20**		**168**
Martinou	EPISKOPI (LEMESOU)	32.88360256	34.6734099	2015	**3**	3																			
Martinou	EPISKOPI (LEMESOU)	32.88360256	34.6734099	2017	**18**	18																			
Martinou	EPISKOPI (LEMESOU)	32.88360256	34.6734099	2018	**118**	118																			
Martinou	EPISKOPI (LEMESOU)	32.88360256	34.6734099	2020	**35**	35																			
Martinou	ATHIENOU	33.54268773	35.0593096	2020	**17**	17																			
Martinou	AKROTIRI	32.96644604	34.60035084	2020	**6**	6																			
Ozbel	MANDRES (LEFKOSIAS)	33.36976699	35.22571666	2018	**207**	197	2				2		1							1		2	2		
Antoniou*	AGIOI TRIMITHIAS	33.23206695	35.11148109	2018	**529**	47		6		3	2									272		33			166
Antoniou*	ANARITA	32.54187123	34.7450802	2013	**52**	5					8	31								4			4		
Antoniou*	PSIMOLOFOU	33.27927353	35.05986102	2013	**15**	4					1												9		1
Antoniou*	SALAMIOU	32.68094708	34.83415058	2013	**101**	6		5			37	33								14			5		1

*A small fraction of these samples was used in the molecular study published by Pavlou et al. (2022).

Pavlou, C., Dokianakis, E., Tsirigotakis, N., Christodoulou, V., Özbel, Y., Antoniou, M., & Poulakakis, N. (2022). A molecular phylogeny and phylogeography of Greek Aegean Island sand flies of the genus Phlebotomus (Diptera: Psychodidae). *Arthropod Systematics & Phylogeny*, *80*, 137–154.
